# Comparison of the Impact of High-Flux Dialysis on Mortality in Hemodialysis Patients with and without Residual Renal Function

**DOI:** 10.1371/journal.pone.0097184

**Published:** 2014-06-06

**Authors:** Hyung Wook Kim, Su-Hyun Kim, Young Ok Kim, Dong Chan Jin, Ho Chul Song, Euy Jin Choi, Yong-Lim Kim, Yon-Su Kim, Shin-Wook Kang, Nam-Ho Kim, Chul Woo Yang, Yong Kyun Kim

**Affiliations:** 1 Department of Internal Medicine, College of Medicine, The Catholic University of Korea, Seoul, Korea; 2 Department of Internal Medicine, College of Medicine, Chung-Ang University, Seoul, Korea; 3 Department of Internal Medicine, Kyungpook National University School of Medicine, Daegu, Korea; 4 Department of Internal Medicine, College of Medicine, Seoul National University, Seoul, Korea; 5 Department of Internal Medicine, College of Medicine, Yonsei University, Seoul, Korea; 6 Department of Internal Medicine, Chonnam National University Medical School, Gwangju, Korea; 7 St. Vincent’s Hospital, Suwon, Korea; 8 MRC for Cell Death Disease Research Center, The Catholic University of Korea, Seoul, Korea; University Medical Center Groningen and University of Groningen, Netherlands

## Abstract

**Background:**

The effect of flux membranes on mortality in hemodialysis (HD) patients is controversial. Residual renal function (RRF) has shown to not only be as a predictor of mortality but also a contributor to β2-microglobulin clearance in HD patients. Our study aimed to determine the interaction of residual renal function with dialyzer membrane flux on mortality in HD patients.

**Methods:**

HD Patients were included from the Clinical Research Center registry for End Stage Renal Disease, a prospective observational cohort study in Korea. Cox proportional hazards regression models were used to study the association between use of high-flux dialysis membranes and all-cause mortality with RRF and without RRF. The primary outcome was all-cause mortality.

**Results:**

This study included 893 patients with 24 h-residual urine volume ≥100 ml (569 and 324 dialyzed using low-flux and high-flux dialysis membranes, respectively) and 913 patients with 24 h-residual urine volume <100 ml (570 and 343 dialyzed using low-flux and high-flux dialysis membranes, respectively). After a median follow-up period of 31 months, mortality was not significantly different between the high and low-flux groups in patients with 24 h-residual urine volume ≥100 ml (HR 0.86, 95% CI, 0.38–1.95, P = 0.723). In patients with 24 h-residual urine volume <100 ml, HD using high-flux dialysis membrane was associated with decreased mortality compared to HD using low-flux dialysis membrane in multivariate analysis (HR 0.40, 95% CI, 0.21–0.78, P = 0.007).

**Conclusions:**

Our data showed that HD using high-flux dialysis membranes had a survival benefit in patients with 24 h-residual urine volume <100 ml, but not in patients with 24 h-residual urine volume ≥100 ml. These findings suggest that high-flux dialysis rather than low-flux dialysis might be considered in HD patients without RRF.

## Introduction

Patients with End-stage renal disease (ESRD) undergoing maintenance hemodialysis (HD) have a high risk of morbidity and mortality [1]. Therefore, the most effective and best-tolerated HD treatment may improve clinical outcomes in this patient population [2]. In particular, the dialyzer used in HD treatment is one of the important determinants of the effectiveness of dialysis. HD using high-flux dialysis membrane can clear more middle molecular weight uremic toxins such as β2-microglobulin than HD using low-flux dialysis membrane because of its higher porosity [3].

Despite the beneficial effects of middle-molecule removal by high-flux dialysis, the effects of dialyzer membrane flux on mortality are controversial. A number of observational studies have suggested that HD using high-flux dialysis membrane results in improved outcomes compared with low-flux dialysis [4]–[7]. However, two large randomized clinical trials, the HEMO study and the European Membrane Permeability Outcome (MPO) study, showed no significant survival difference between HD using high-flux dialysis membrane and HD using low-flux dialysis membrane [8], [9]. This discrepancy may be due to differences in the populations studied or study design.

Residual renal function is known to be an important determinant of serum β2-microglobulin level and a contributor of β2-microglobulin clearance in patients with HD [10], [11]. In patients with greater residual renal function, the beneficial effects of β2-microglobulin removal by high-flux dialysis may not be apparent. Therefore, it may be postulated that the beneficial effect of high-flux dialysis on mortality may be different between patients with different degrees of residual renal function.

This study aimed to determine the interaction of residual renal function with dialyzer membrane flux on mortality in patients enrolled in the Clinical Research Center (CRC) registry for ESRD cohort which is an observational prospective cohort study conducted in Korea.

## Materials and Methods

### Study Population

All patients included in this study were enrolled in the CRC registry for ESRD. This is an ongoing observational prospective cohort study patients with ESRD from 31 medical centers in Korea. The cohort started in April 2009 and included adult (>18 years of age) dialysis patients. A total 3,067 patients undergoing HD were enrolled in this cohort. For the present study, we excluded patients for whom information about the dialysis membrane used or 24 h-urine volume was not available (n = 1,261). So, 1,806 patients were included in the final analysis.

Demographic and clinical data were collected at the time of enrollment. Assessment of dialysis characteristics and measurements of health were done every 6 months until follow-up was complete. Dates and causes of mortality were reported throughout the follow-up period.

### Ethics

The study was approved by the institutional review board at each center [The Catholic University of Korea, Bucheon St. Mary’s Hospital; The Catholic University of Korea, Incheon St. Mary’s Hospital; The Catholic University of Korea, Seoul St. Mary’s Hospital; The Catholic University of Korea, St. Mary’s Hospital; The Catholic University of Korea, St. Vincent’s Hospital; The Catholic University of Korea, Uijeongbu St. Mary’s Hospital; Cheju Halla General Hospital; Chonbuk National University Hospital; Chonnam National University Hospital; Chung-Ang University Medical Center; Chungbuk National University Hospital; Chungnam National University Hospital; Dong-A University Medical Center; Ehwa Womens University Medical Center; Fatima Hospital, Daegu; Gachon University Gil Medical Center; Inje University Pusan Paik Hospital; Kyungpook National University Hospital; Kwandong University College of Medicine, Myongji Hospital; National Health Insurance Corporation Ilsan Hospital; National Medical Center; Pusan National University Hospital; Samsung Medical Center, Seoul; Seoul Metropolitan Government, Seoul National University, Boramae Medical Center; Seoul National University Hospital; Seoul National University, Bundang Hospital; Yeungnam University Medical Center; Yonsei University, Severance Hospital; Yonsei University, Gangnam Severance Hospital; Ulsan University Hospital; Wonju Christian Hospital (in alphabetical order)] and performed in accordance to the Declaration of Helsinki. Written informed consent was obtained from all patients.

### Clinical and Dialysis Parameters

In the CRC registry for ESRD study, baseline demographic and clinical data including age, sex, body mass index (BMI), type of dialysis membrane, primary causes of ESRD, comorbidities (cardiovascular disease and DM), laboratory values, and therapeutic characteristics were recorded. Cardiovascular disease was defined as the presence of coronary artery disease, congestive heart failure, peripheral vascular disease, cerebrovascular disease, or atrial fibrillation. Serum hemoglobin, serum albumin, serum creatinine, blood urea nitrogen, serum potassium and serum total cholesterol (TC) were measured. The single-pool Kt/V (spKt/V) was determined by two-point urea modeling based on the intradialytic reduction in blood urea and intradialytic weight loss. Timed 24 h urine collection was performed during the interdialytic intervals at the time of enrollment and 24 h-urine volume was recorded. Zero- residual renal function was operationally defined as having 24 h-urine volume <100 ml. Patients were grouped as having zero- residual renal function or non-zero residual renal function. To estimate the residual glomerular filtration rate, residual renal clearance (ml/min) was calculated as the mean of the creatinine clearance and urea clearance in patients with 24 h-urine volume ≥100 ml [12].

In order to analyze the effects of the dialysis membrane type on mortality in patients with zero- and non-zero residual renal function, the patients were further divided into high-flux and low-flux dialysis groups according to the type of dialysis membrane used. High-flux dialysis was defined as an ultrafiltration coefficient of ≥20 ml/mm Hg per hour and a sieving coefficient for β2-microglobulin >0.6. Low-flux dialysis was defined as an ultrafiltration coefficient of ≤10 ml/mm Hg per hour and a sieving coefficient for β2-microglobulin = 0 [9].

A total of 21 types of low-flux dialyzer and 26 types of high-flux dialyzers were used in this study. The most common low-flux dialyzer was the Gambro polyflux 14L (used in 34.2% of cases) and the most common high-flux dialyzer was the Gambro polyflux 170H (used in 18.9% of cases). All of the dialysis membrane materials were synthetic membranes in the high-flux dialyzer group, whereas 99.0% contained synthetic membranes and 1.0% contained substituted cellulose membranes in low-flux dialyzer group. All dialysis sessions were performed without reuse of the dialyzers. All dialysate solutions were bicarbonate-based. Dialysate complied with the criteria adopted by the European Best Practice Guidelines [13] and the ultrapure dialysates were used in all patients using high-flux dialyzer.

### Outcomes

The clinical outcome of this study was all-cause mortality. For each death, the principal investigator at that given institution completed a form that included cause of death according to the CRC registry for ESRD study classification.

### Statistical Analyses

Data with continuous variables and normal distribution are presented as means ± SD, and those without normal distribution are presented as the median with ranges as appropriate for the type of variable. Student’s t-tests, Mann–Whitney U tests, one-way ANOVA tests and Kruskal-Wallis tests were used to determine the differences in continuous variables. Categorical variables are presented as percentages. Pearson’s chi-square test or Fisher’s exact test were used to determine the differences in categorical variables.

Absolute mortality rates were calculated per 100 person-years of follow-up. The survival curves were estimated using the Kaplan–Meier method and compared by the log-rank tests between the high- and low-flux dialysis groups. The Cox proportional hazard regression model was used to calculate hazard ratio (HR) with 95% confidence interval (CI) for all-cause mortality. Analyses were adjusted for potential confounders including age, gender, use of high-flux membrane, BMI, diabetes mellitus, cardiovascular disease, primary cause of ESRD, duration of dialysis therapy, systolic blood pressure (BP), diastolic BP, hemoglobin, serum albumin, serum β2-microglobulin, serum TC, type of vascular access, residual renal clearance, and spKt/V. A value of P<0.05 was considered to be statistically significant. All statistical analyses were performed using SPSS 11.5 software (Chicago, IL, USA).

## Results

### Patients Characteristics

A total of 893 patients with 24 h-residual urine volume ≥100 ml and 913 patients with 24 h-residual urine volume <100 ml were included in this study. Table 1 shows the baseline characteristics of participants.

**Table 1:** Baseline characteristics of the study populations.

Characteristics	24 h-residual urine volume ≥100 ml(n = 893)			24 h-residual urine volume <100 ml(n = 913)		
	Low-Flux(n = 569)	High-Flux(n = 324)	p	Low-Flux(n = 570)	High-Flux(n = 343)	p
Age (years)	59±14	58±13	0.504	59±13	57±13	0.027
Male, n (%)	338 (9.4)	205 (63.3)	0.255	331 (58.1)	184 (53.6)	0.192
Body mass index (kg/m^2^)	23.0±3.7	22.6±3.1	0.082	22.6±3.3	22.2±3.5	0.137
Comorbidities						
Diabetes mellitus, n (%)	303 (53.3)	192 (59.3)	0.108	339 (59.5)	165 (48.1)	0.001
Cardiovascular diseases, n (%)	207 (36.4)	144 (44.4)	0.029	253 (44.4)	146 (42.6)	0.865
Causes of ESRD, n (%)			0.059			0.001
Diabetes mellitus	302 (53.1)	189 (58.3)		300 (52.6)	150 (43.7)	
Glomerulonephritis	89 (15.6)	40 (12.3)		83 (14.6)	49 (14.3)	
Renal vascular disease	99 (17.4)	44 (13.6)		74 (13.0)	48 (22.7)	
Others/unknown	79 (13.9)	51 (15.7)		113 (19.8)	66 (19.2)	
Duration of dialysis therapy (months)	0 (0–3)	0 (0–7)	0.114	17 (0–53)	46 (17–84)	<0.001
Measurement before dialysis						
Systolic BP (mmHg)	141±24	143±25	0.233	142±21	143±20	0.438
Diastolic BP (mmHg)	77±14	76±14	0.278	79±13	80±12	0.073
Hemoglobin (g/dl)	9.0±1.7	9.2±1.7	0.060	10.1±1.5	10.3±1.4	0.071
Serum albumin (g/dl)	3.5±0.6	3.4±0.6	0.067	3.7±0.6	3.9±0.5	<0.001
Serum TC (mg/dl)	154±43	154±47	0.988	158±44	155±37	0.275
Serum β2-microglobulin (mg/L)	22.2 (17.2–29.3)	20.3 (15.4–26.2)	0.011	34.4 (23.0–44.9)	30.0 (24.0–44.3)	0.788
Vascular access			<0.001			<0.001
Arteriovenous fistula, n (%)	174 (30.9)	139 (43.6)		330 (58.8)	247 (74.2)	
Arteriovenous graft, n (%)	49 (8.7)	35 (11.0)		98 (17.5)	47 (14.1)	
Catheter, n (%)	340 (60.4)	145 (45.5)		133 (23.7)	39 (11.7)	
24 h-urine volume (ml)	900 (518–1300)	810 (465–1300)	0.285	0 (0–0)	0 (0–0)	0.829
Residual renal clearance (ml/min)	2.9 (1.4–5.2)	2.7 (1.4–4.2)	0.062	-	-	
spKt/V	1.43±0.66	1.46±0.44	0.539	1.52±0.39	1.35±0.78	0.001

Data are expressed as mean ± SD or medians (interquartile percentile) or numbers (percentages).

Abbreviations: ESRD, end-stage renal disease; BP, blood pressure; TC, total cholesterol; Kt/V: K, dialyzer clearance; t, time; V, volume of water a patient’s body contains.

In the patients with 24 h-residual urine volume ≥100 ml, 64% (569 of 893) patients were dialyzed using low-flux dialysis membranes and 36% (324 of 893) were dialyzed using high-flux dialysis membranes. The high-flux group had a higher prevalence of cardiovascular diseases and lower serum β2-microglobulin levels than the low-flux group. Arteriovenous fistula as the vascular access was more used in high-flux group and catheter was more used in low-flux group. There was no significant difference in rate of arteriovenous graft use between the high- and the low-flux groups. There were no significant differences in age, gender, BMI, prevalence of diabetes mellitus, primary cause of ESRD, duration of dialysis therapy, systolic BP, diastolic BP, serum hemoglobin levels, serum albumin levels, serum TC levels, 24-h urine volume, residual renal clearance and spKt/V between the high- and the low-flux groups.

In the patients with 24 h-residual urine volume <100 ml, 62% (570 of 913) were dialyzed using low-flux dialysis membranes and 38% (343 of 913) were dialyzed using high-flux dialysis membranes. The high-flux group was younger and had a lower prevalence of diabetes mellitus. Diabetes mellitus as a primary cause of ESRD was more prevalent in lower flux group. The high-flux group had a longer duration of dialysis therapy, higher serum albumin levels and lower spKt/V than the low-flux group. Arteriovenous fistula as the vascular access was more used in high-flux group and catheter was more used in low-flux group. There was no significant difference in rate of arteriovenous graft use between the high- and the low-flux groups. There were no significant differences in gender, BMI, prevalence of cardiovascular diseases, systolic BP, diastolic BP, serum hemoglobin levels, serum TC levels, serum β2-microglobulin level and 24-h urine volume between the high- and the low-flux groups.

### Effect of Membrane Flux on All-cause Mortality

The median follow-up period was 31 months (interquartile range, 13–40 months). Total of 170 deaths occurred during the follow-up period. Cardiovascular diseases were the leading cause of death (32.9% of all deaths). Table 2 shows the causes of death in each group. The distribution of causes of death was not significantly different between high- and low-flux groups in patients with 24 h-residual urine volume ≥100 ml or in patients with 24 h-residual urine volume <100 ml (p = 0.239 and p = 0.938, respectively).

**Table 2:** Causes of deaths in each group.

	24 h-residual urinevolume ≥100 ml		24 h-residual urinevolume <100 ml	
	Low-Flux(44 deaths)	High-Flux(25 deaths)	Low-Flux(75 deaths)	High-Flux(26 deaths)
Cardiovascular diseases includingcerebrovascular diseases, n (%)	13 (29.5)	12 (48.0)	23 (30.7)	8 (30.8)
Infectious diseases, n (%)	14 (31.8)	2 (8.0)	24 (32.0)	7 (26.9)
Malignancy, n (%)	2 (4.5)	1 (4.0)	7 (9.3)	2 (7.7)
Others, n	15 (34.1)	10 (40.0)	21 (28.0)	9 (34.6)

**Figure 1:**
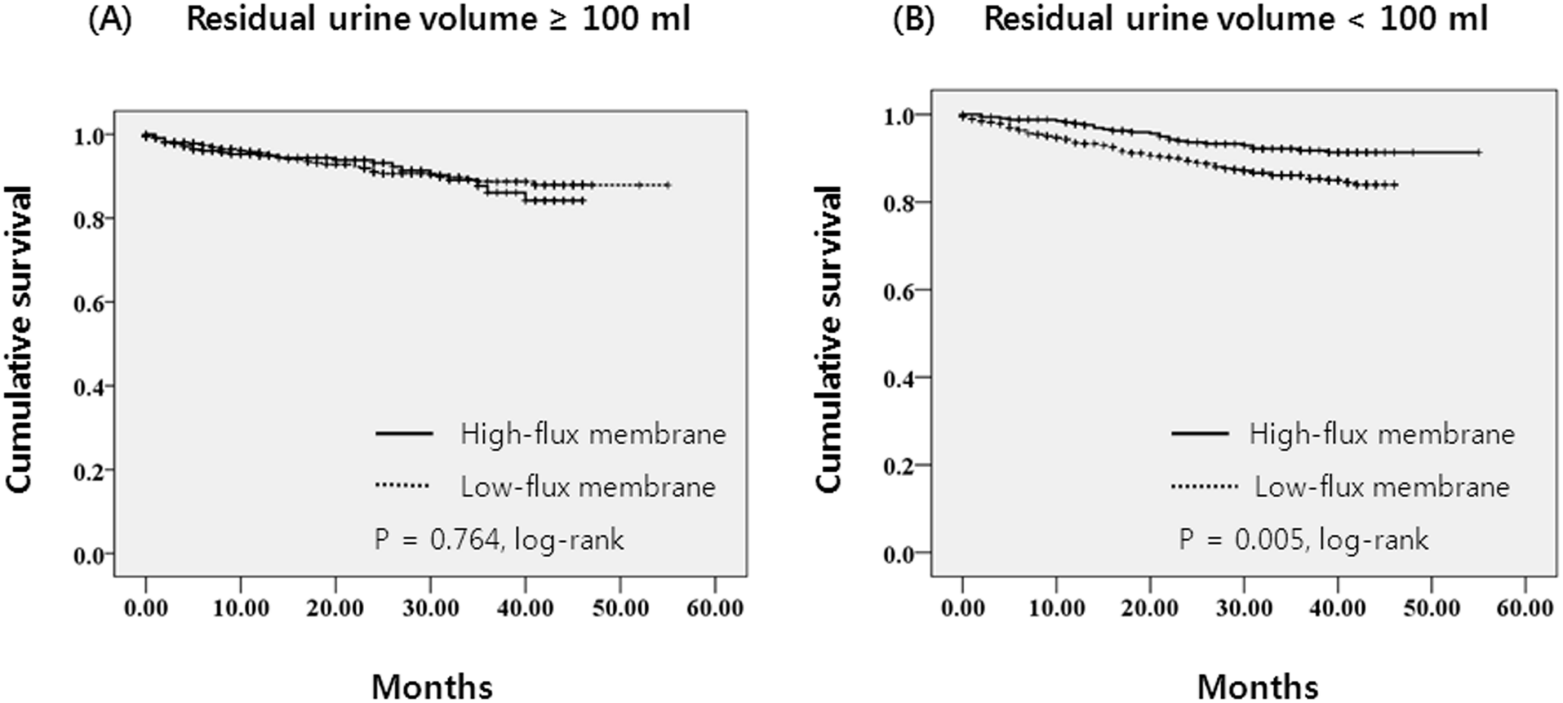
Kaplan-Meier survival curve for mortality in (A) patients with 24 h-residual urine volume ≥100 ml (P = 0.764 by log-rank test) and in (B) patients with 24 h-residual urine volume <100 ml (P = 0.005 by log-rank test).

In patients with 24 h-residual urine volume ≥100 ml, 197 patients left the study during the follow-up period for reasons other than death, including 38 patients who received kidney transplantation, 81 patients who transferred to a nonparticipating hospital, 44 patients who refused further participation and 34 for unspecified reasons. There were 69 deaths in this group during the follow-up period. The leading cause of death was cardiovascular diseases (36.2% of all deaths) (Table 2). The absolute mortality rate during the follow-up period was 4.2 deaths per 100 person-years. In univariate Cox regression analysis, use of high-flux membrane was not associated with mortality (HR 1.08, 95% CI, 0.66–1.76, P = 0.765). Figure 1A shows the Kaplan-Meier plot for all-cause mortality in the high- and the low-flux groups for the patients with 24 h-residual urine volume ≥100 ml. As shown, there was no significant difference in survival between the high-flux group and the low-flux group (P = 0.764 by log-rank test). After adjustment for demographics, comorbid conditions, and laboratory data, the adjusted HR for mortality in the high-flux group was 0.86 (95% CI, 0.38–1.95, P = 0.723), implying the mortality was not significantly different between the high- and the low-flux groups in the patients with 24 h-residual urine volume ≥100 ml (Table 3).

**Table 3:** Multivariate Cox regression analysis of mortality in study populations.

	24 h-residual urine volume ≥100 ml			24 h-residual urine volume <100 ml		
	HR	95% CI	P	HR	95% CI	P
High-flux membrane (versus low-flux)	0.86	0.38–1.95	0.723	0.40	0.21–0.78	0.007
Age (1-yearincrement)	1.09	1.05–1.14	<0.001	1.04	1.01–1.07	0.005
Male (versus female)	0.72	0.32–1.64	0.431	0.86	0.45–1.62	0.633
BMI (per increment of 1 kg/m^2^)	0.98	0.87–1.10	0.743	0.95	0.86–1.05	0.325
Comorbidities						
Diabetes mellitus (versus none)	0.85	0.12–5.88	0.869	2.19	0.73–6.57	0.162
Cardiovascular diseases (versus none)	1.54	0.74–3.22	0.250	1.28	0.69–2.36	0.437
Causes of ESRD						
Diabetes mellitus (versus non diabetes mellitus)	1.32	0.19–9.23	0.78	2.01	0.71–5.89	0.183
Systolic BP (per increment of 10 mmHg)	1.00	0.98–1.02	0.998	1.01	0.99–1.03	0.276
Diastolic BP (per increment of 10 mmHg)	1.02	0.98–1.06	0.385	1.00	0.97–1.04	0.858
Hemoglobin (per increment of 1 g/dl)	0.86	0.67–1.11	0.256	1.05	0.85–1.30	0.645
Serum albumin (per increment of 1 g/dl)	0.41	0.20–0.82	0.012	0.81	0.41–1.61	0.552
Serum β2-microglobulin ((per increment of 1 mg/L)	1.00	1.00–1.00	0.801	1.00	0.99–1.01	0.769
Serum TC (per increment of 10 mg/dl)	1.00	0.99–1.01	0.993	1.00	0.99–1.01	0.686
Residual renal clearance (per increment of 1 ml/min)	1.10	0.97–1.25	0.131			
spKt/V	1.15	0.51–2.62	0.735	0.57	0.31–1.04	0.067

Multivariate model includes age, gender, use of high-flux membrane, BMI, diabetes mellitus, cardiovascular disease, causes of ESRD, duration of dialysis therapy, systolic BP, diastolic BP, hemoglobin, serum albumin, serum β2-microglobulin, serum TC, type of vascular access, residual renal clearance and spKt/V.

Abbreviations: BMI, body mass index; HR, hazard ratio; TC, total cholesterol.

In patients with 24 h-residual urine volume <100 ml, 174 patients left the study during the follow-up period for reasons other than death, including 51 patients who received kidney transplantation, 78 patients who transferred to a nonparticipating hospital, 21 patients who refused further participation and 24 for unspecified reasons. There were a total of 101 deaths in this group during the follow-up period. The leading causes of death were cardiovascular diseases (30.7% of all deaths) and infectious diseases (30.7%of all deaths) (Table 2). The absolute mortality rate during the follow-up period was 4.2 deaths per 100 person-years. In univariate Cox regression analysis, the high-flux group was significantly associated with reduced mortality (HR 0.53, 95% CI, 0.34–0.83, P = 0.005). Figure 1B shows the Kaplan-Meier plot for all-cause mortality in the high- and the low-flux groups in patients with 24 h-residual urine volume <100 ml. Survival was increased in the high-flux group compared to the low-flux group (P = 0.005 by log-rank test). Even after adjustment for demographics, comorbid conditions, and laboratory data, the adjusted HR for mortality in the high-flux group was 0.40 (95% CI, 0.21–0.78, P = 0.007), implying that the high-flux group had a risk of death that was 60% lower than the low-flux group in patients with 24 h-residual urine volume <100 ml (Table 3).

## Discussion

The major findings of this study were that HD using high-flux dialysis membrane was associated with decreased mortality compared to HD using low-flux dialysis membrane in patients with 24 h-residual urine volume <100 ml, whereas there was no significant difference in mortality between the high- and the low-flux dialysis group in patients with 24 h-residual urine volume ≥100 ml. These data suggest that high-flux dialysis impacts survival differently according to residual renal function and that high-flux dialysis is superior to low-flux dialysis in patients without residual renal function.

Residual renal function is associated with improved survival and clinical outcomes such as hospitalization, nutrition, anemia, and serum phosphorous control in HD patients [14]–[16]. Furthermore, residual renal function is a strong predictor of serum β2-microglobulin levels, since the kidney is the primary organ for the clearance of β2-microglobulin [11]. In this study, serum β2-microglobulin levels were significantly higher in patients with 24 h-residual urine volume <100 ml (31 mg/L, interquartile range, 23–45 mg/L) than in those with 24 h-residual urine volume ≥100 ml (21 mg/L, interquartile range, 16–28 mg/L) (p<0.001) (data not shown). A previous study also reported that increment of residual renal function was associated with a decrease in serum β2-microglobulin levels in dependent of years on dialysis [11]. Therefore, the beneficial effects of high-flux dialysis by clearance of middle molecules such as β2-microglobulin on clinical outcomes may be overshadowed by residual renal function in HD patients. Thus, it could be postulated that the beneficial effect of high-flux dialysis may be more apparent in patients with lesser residual renal function.

The HEMO study showed that there was no statistically significant interaction between baseline residual renal function and the type of flux intervention with respect to all-cause mortality although there was a trend towards decreased mortality in patients with lesser residual renal function [17]. They reported that all-cause mortality rates were not significantly different between patients with residual urea clearance ≤0.24 ml/min and those with residual urea clearance >0.24 ml/min (P = 0.24) [17], which is not consistent with our results. There are a number of possible explanations for this discrepancy.

First, it should be noted that the HEMO study only included patients with residual urea clearance <1.5 ml/min/35L of urea. Because the impact of high-flux dialysis on mortality may be less apparent in patients with greater residual renal function, the exclusion of patients with greater residual renal function may be a confounding factor in comparing the impact of high-flux dialysis on mortality according to residual renal function.

Additionally, the HEMO study included HD patients in which dialyzers were reused. Although the relationship between reuse of the dialyzer and effectiveness of removal of middle molecules has been controversial, reuse of dialyzers may be associated with structural damage of the membrane and a reduced permeability to middle molecules [18], [19]. Therefore, reuse of dialyzer also may be a confounder to determine the impact of dialyzer membrane flux on mortality.

Another large randomized controlled trial, the MPO study, showed that there was no significant difference in mortality between high- and low-flux dialysis in the whole cohort [9]. In subgroup analysis, the MPO study showed a survival benefit with high-flux dialysis in patients with serum albumin level ≤4 mg/dl, while there was no significant difference in mortality between high- and low-flux dialysis in patients with serum albumin level >4 mg/dl. Data on interaction between residual renal function and the type of flux intervention with respect to all-cause mortality were not shown in the MPO study. However, the MPO study provides some interesting clues on the impact of residual renal function on the relationship between high flux dialysis and mortality. First, patients with serum albumin level ≤4 mg/dl in the MPO study had longer duration of follow-up than the patients with serum albumin level >4 mg/dl because the study protocol was amended during the course of the study [9]. The longer duration of follow-up in patients with serum albumin level ≤4 mg/dl may explain the relationship between survival benefit with high-flux dialysis and residual renal function. Long duration of dialysis may cause accumulation of toxic middle molecules and decrease residual renal function to remove them. Therefore, in the MPO study, the patients with serum albumin level ≤4 mg/dl could have benefited more from the removal of toxic middle molecules with high-flux dialysis than the patients with serum albumin level >4 mg/dl because of the longer follow up. Second, the survival benefit of high-flux dialysis in the patients with serum albumin level ≤4 mg/dl was evident only after about 12 months of follow-up period, possibly when the residual renal function was lost. These findings of the MPO study therefore support the results of our study.

Our findings have a number of clinical implications. The European Best Practice Guideline (EBPG) relating to dialyzer membrane permeability recommends that the use of synthetic high-flux membranes should be considered to delay long-term complications of HD therapy [20]. The EBPG suggests the specific indication for high-flux dialysis to reduce dialysis-related amyloidosis, to improve control of hyperphosphatemia, to reduce the increased cardiovascular risk, and to improve control of anemia [20]. Our findings support the evidence for use of high-flux dialysis membrane and further contribute to the indications established for high-flux dialysis therapy in HD patients without residual renal function.

Our study has several limitations. First, the design of our study was not a randomized, controlled study but rather was a prospective observational study. The prescription of the dialyzer might be influenced by the results of previous study for the membrane flux on mortality such as MPO study. Accordingly, some baseline characteristics between the high-flux dialysis group and low-flux dialysis group differed in our study, indicating potential selection bias. In addition, the median follow-up period of 31 months was relatively short. Finally, despite the multicenter nature of the study, our cohort consisted of solely Korean patients. Therefore, it is uncertain whether our results can be generalized to other ethnic groups with HD treatment.

In conclusion, we found that HD using high-flux dialysis membranes had survival benefit in patients with 24 h-residual urine volume <100 ml, but not in patients with 24 h-residual urine volume ≥100 ml. These findings suggest that dialyzer membrane flux impacts survival differently according to residual renal function. Thus, high-flux dialysis rather than low-flux dialysis might be considered in HD patients without residual renal function.
